# *Pseudomonas putida* rDNA is a favored site for the expression of biosynthetic genes

**DOI:** 10.1038/s41598-019-43405-1

**Published:** 2019-05-07

**Authors:** Andreas Domröse, Jennifer Hage-Hülsmann, Stephan Thies, Robin Weihmann, Luzie Kruse, Maike Otto, Nick Wierckx, Karl-Erich Jaeger, Thomas Drepper, Anita Loeschcke

**Affiliations:** 1 Institute of Molecular Enzyme Technology, Heinrich Heine University Düsseldorf, Forschungszentrum Jülich, Jülich, D-52425 Germany; 2 Cluster of Excellence on Plant Sciences (CEPLAS), Heinrich Heine University Düsseldorf, Düsseldorf, D-40225 Germany; 3 Bioeconomy Science Center (BioSC), Forschungszentrum Jülich, Jülich, D-52425 Germany; 4 Institute of Applied Microbiology (iAMB), Aachen Biology and Biotechnology (ABBt), RWTH Aachen University, Aachen, D-52074 Germany; 5 Institute of Bio- and Geosciences (IBG-1), Forschungszentrum Jülich, Jülich, D-52425 Germany

**Keywords:** Expression systems, Applied microbiology

## Abstract

Since high-value bacterial secondary metabolites, including antibiotics, are often naturally produced in only low amounts, their efficient biosynthesis typically requires the transfer of entire metabolic pathways into suitable bacterial hosts like *Pseudomonas putida*. Stable maintenance and sufficient expression of heterologous pathway-encoding genes in host microbes, however, still remain key challenges. In this study, the 21 kb prodigiosin gene cluster from *Serratia marcescens* was used as a reporter to identify genomic sites in *P. putida* KT2440 especially suitable for maintenance and expression of pathway genes. After generation of a strain library by random Tn5 transposon-based chromosomal integration of the cluster, 50 strains exhibited strong prodigiosin production. Remarkably, chromosomal integration sites were exclusively identified in the seven rRNA-encoding *rrn* operons of *P. putida*. We could further demonstrate that prodigiosin production was mainly dependent on (i) the individual *rrn* operon where the gene cluster was inserted as well as (ii) the distance between the *rrn* promoter and the inserted prodigiosin biosynthetic genes. In addition, the recombinant strains showed high stability upon subculturing for many generations. Consequently, our findings demonstrate the general applicability of rDNA loci as chromosomal integration sites for gene cluster expression and recombinant pathway implementation in *P. putida* KT2440.

## Introduction

Microbial natural products are an invaluable source of bioactive compounds such as antibiotics. Since these high-value metabolites are often naturally produced in only low amounts or the natural producers are not amenable to genetic engineering and bioprocess development, efficient biosynthesis of these compounds, enabling studies e.g. on their bioactivities or the underlying biocatalytic reactions, typically requires the transfer of entire metabolic pathways into heterologous expression hosts^1^.

The Gram-negative γ-proteobacterium *Pseudomonas putida* is especially suited for heterologous secondary metabolite biosynthesis, because its metabolism is supportive of various natural product pathways and conveys broad tolerance to compounds which are toxic to other bacteria^2,3^. Therefore, multiple tools including vector and promoter sets have been developed to enable functional gene expression in this host^4–6^. In addition, since the stable maintenance and strong expression of pathway-encoding gene clusters still represent major bottlenecks for effective production^7^, the chromosomal integration of target genes is gaining more and more interest^8,9^.

In our previous studies, we established the pathway transfer and expression (TREX) system, which allows the straightforward implementation of heterologous secondary metabolite pathways in different bacterial species. TREX employs the random chromosomal integration of a target gene cluster by Tn5-based transposition, followed by the concerted expression of all biosynthetic genes. This can be realized by use of T7 RNA polymerase-dependent promoters^10^ or an intrinsically strong chromosomal promoter like those driving expression of the ribosomal RNA-encoding genes of *P. putida* KT2440^11,12^. Chromosomal insertion of foreign biosynthetic genes downstream of the latter may be especially favorable for the functional expression of a biosynthetic pathway because this locus possesses two specific characteristics: (i) In *P. putida* KT2440, the ribosomal RNA, i.e. 5S, 16S and 23S rRNA, is encoded in seven rRNA operons, also referred to as *rrn* operons or rDNA, across the bacterial chromosome^13,14^. Although rRNA generation is essential for maintaining cellular life, the insertion of genes in one *rrn* operon copy is not deleterious due to the gene redundancy^11,15^. (ii) At the same time, the promoters of *rrn* operons are commonly regarded as highly active^16^. This may be particularly relevant in the implementation of transcription levels required for the functional expression of large gene clusters since the likelihood of eventual (Rho mediated) RNA polymerase dissociation^17^ from the template increases with the transcript length. Together with mRNA degradation at the 3′ terminus^18,19^, this can cause low transcript levels of distal genes. A strong promoter may help to maintain sufficient transcript levels of genes at the end of the transcriptional unit, while the 3′ fusion of the mRNA with the highly stable rRNA could decrease the rate of degradation. Such chromosomal loci can thus be of particular relevance for the implementation of a heterologous biosynthetic pathway in *P. putida*, and can be identified e.g. by Tn5 transposition.

In the present study, we intended to further pursue this approach in order to identify additional chromosomal loci of *P. putida* suitable for the stable integration and strong expression of clustered biosynthetic genes by a native promoter. To this end, the 21 kb prodigiosin gene cluster (*pig*) from *Serratia marcescens* was used as a ‘reporter gene cluster’ because functional expression of all cluster genes in *P. putida* results in a red phenotype that can easily be screened for by visual inspection. To search for suitable genomic loci within the entire chromosome of *P. putida* KT2440, we created a *pig* strain library by applying genomic integration of the pathway genes *via* Tn5-based random transposition. Based on intense red coloration phenotypes, a set of 50 strains synthesizing the pigment was identified and analyzed with respect to individual chromosomal insertion loci and corresponding prodigiosin production titers. Remarkably, in all of the analyzed *P. putida* strains, the *pig* cluster was integrated in one of the seven *rrn* operons. Prodigiosin levels were strongly influenced by the specific *rrn* operon copy in which the biosynthetic genes were inserted and further modulated by the distance between the *rrn* promoter and inserted *pig* genes. This study therefore demonstrates the applicability of the *P. putida rrn* operons as particularly suitable gene cluster integration sites potentially allowing the identification and synthesis of new and valuable natural products in this versatile production host.

## Results

### Generation of a *P. putida* TREX-*pig* strain library

In order to identify locations in the chromosome of *P. putida* KT2440 which allow the activation of a heterologous biosynthetic pathway – a process that includes the stable insertion and expression of multiple pathway genes – we employed transposon Tn5-based integration of the prodigiosin gene cluster (*pig*) from *S. marcescens*. Transposon Tn5 is a widely established tool for untargeted gene integration into the bacterial chromosome and permits an almost completely unbiased sampling of the chromosomal space^20–22^. The *pig* gene cluster is particularly suited as a ‘reporter gene cluster’ for the following reasons: (i) The *pigA-N* gene cluster comprises 14 genes spanning 21 kb and can thus be regarded as a representative of a multitude of mid-sized biosynthetic gene clusters; it was thus preferred over a common reporter gene like GFP. (ii) The *pig* genes do not exhibit any sequence similarity to the genome of *P. putida* KT2440 that could cause homologous recombination events and would compromise random Tn5 integration into the chromosome. (iii) The gene cluster consists of unidirectionally oriented biosynthetic genes naturally organized in one transcriptional unit^23,24^, thereby allowing transcription of all genes by a single chromosomal promoter. (vi) All pathway-associated enzymes can be functionally expressed in *P. putida* and the activation of the prodigiosin biosynthesis in *P. putida* results in the formation of the red pigment which can be applied as easily quantifiable production readout^11,12^.

**Figure 1:**
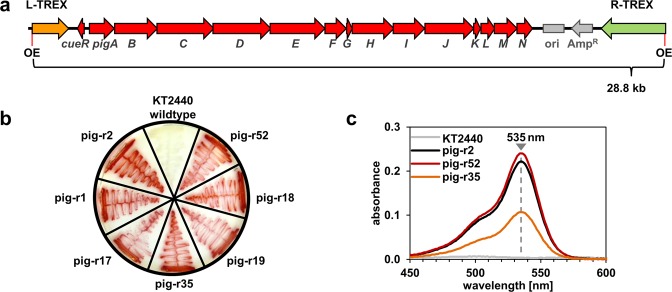
Phenotypes of *P. putida* pig-r strains with a chromosomally inserted TREX-*pig* transposon. (**a**) Schematic representation of the TREX-*pig* transposon, which was transferred on previously established plasmid pTREX-pig to *P. putida* KT2440. The prodigiosin biosynthetic genes from *S. marcescens* and the TREX cassettes including the outer ends (OE) of the transposon Tn5 are indicated. (**b**) Fifty *P. putida* strains including previously established pig-r1 and pig-r2 were selected for further characterization based on their intense color phenotype, exemplarily shown for strains pig-r52, -r18, -r19, -r35 and -r17. The wildtype *P. putida* KT2240 is shown for reference. (**c**) Representative absorbance spectra of cell extracts from *P. putida* pig-r35 and -r52, showing the typical prodigiosin absorption maximum at 535 nm like previously established strain pig-r2.

We used the previously constructed plasmid pTREX-*pig* for conjugational transfer of *pig* genes to *P. putida* KT2440. The plasmid carries the complete prodigiosin gene cluster *pigA-N* and the adjacent gene *cueR*, which is involved in the intrinsic regulation cascade of *S. marcescens* but is of minor importance for this study (Fig. 1a). The gene cluster thus also includes the native promoter region of the *pigA-N* operon; however, when transferred into the heterologous host *P. putida* and thus in the absence of the complex native regulatory network^25,26^, it is not functional^10^. The biosynthetic gene cluster is flanked by the two TREX DNA cassettes which include a gentamicin resistance gene (in R-TREX) as well as the elements of transposon Tn5, enabling random chromosomal integration^10,11^. To integrate the *pig* gene cluster at potent expression sites distributed across the host’s chromosome, we followed a workflow previously established for generating *P. putida* prodigiosin production strains^11,12^. A library of ca. 50,000 *P. putida* clones carrying the TREX-*pig* transposon was generated and screened for clones exhibiting intense red coloration as an indicator of *pig* gene expression. The total number of screened clones ensured theoretical coverage of the *P. putida* chromosome comprising 5729 annotated genes with insertions ca. every 248 bp in both strands of the chromosome, assuming that transposition is completely unbiased. We found 50 clones, representing 0.1% of the total number of library clones, showing expression of the prodigiosin pathway indicated by an intense red phenotype, which were designated *P. putida* pig-r3 to pig-r52 and re-streaked to comparatively evaluate the color phenotype (Fig. 1b). All strains exhibited a very intense red pigmentation except strains pig-r33 and pig-r36, which showed substantially weaker coloration and were therefore excluded from further analysis. In order to corroborate prodigiosin biosynthesis in the new pig-r strains, absorbance spectra of cell extracts derived from representative strains were analyzed after small scale cultivation in liquid TB medium (Fig. 1c). The spectra exhibited an absorption maximum at 535 nm which is typical for prodigiosin^11^ and indicated that the colored phenotype was indeed caused by accumulation of prodigiosin. In addition, differences in the intensity of the signal gave a first indication that the strains exhibited differential production characteristics. The previously constructed strains *P. putida* pig-r1 and pig-r2^11^ and the here newly generated strains added up to a set of 50 prodigiosin producing strains that were subjected to detailed analysis of insertion loci and prodigiosin production.

### Identification of the *rrn* operons as insertion loci of *pig* genes in all pig-r strains

**Figure 2:**
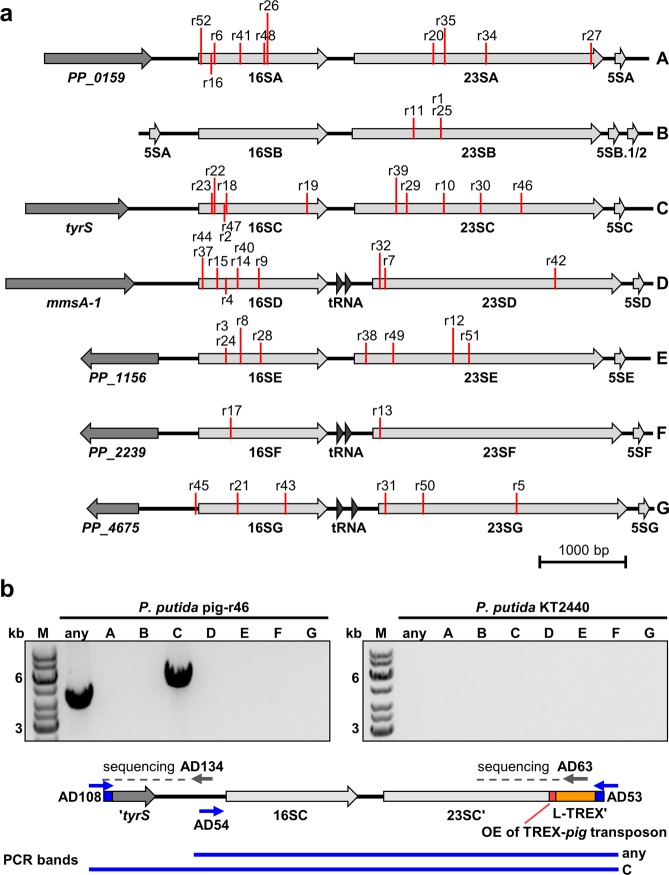
Insertion loci of the TREX-*pig* transposon within the *rrn* operons of *P. putida* pig-r1 to pig-r52. (**a**) The seven rRNA-encoding *rrn* operons (A–G) of *P. putida* KT2440 are shown together with the different genes upstream of the *rrn* promoter regions. The insertion loci of the TREX-*pig* transposon in the *P. putida* strains pig-r1 to pig-r52 are indicated. (**b**) Determination of TREX-*pig* transposon insertion sites, exemplarily shown for strain *P. putida* pig-r46. After PCR product formation with primers AD54 and AD53 indicated transposon insertion into one of the *rrn* operons (any), seven specific forward primers including AD108 for *rrnC* were used (for details see Supplementary Fig. S1). Here, only primer AD108 which specifically binds to the *tyrS* sequence, together with AD53, generated a PCR product, indicating transposon integration in *rrnC*. Sequencing from primer AD134 verified the assignment to *rrnC* and sequencing from primer AD63 identified the exact position of the TREX-*pig* transposon within the *rrn* operon.

Since we found in our previous study the *pig* genes to be inserted into the rDNA of *P. putida* pig-r1 and pig-r2, we first investigated if this was likewise the case for any of the 48 newly generated pig-r strains. To this end, we performed a PCR screen of all strains using a primer pair that would generate a PCR product in case the TREX-*pig* transposon was inserted in one of the seven rRNA-encoding *rrn* operons of *P. putida* KT2440. The seven operons, which are designated *rrnA* to *rrnG*, are similarly structured (Fig. 2a) and the promoters as well as the 16S rRNA (1.5 kb) and 23S rRNA (2.9 kb) genes show very high sequence identities (see Supplementary Table S1 for similarity matrices), which are in all cases followed by likewise conserved 5S rRNA genes (Fig. 2a). The operons D, F and G additionally feature tRNA-coding sequences between the 16S and 23S rRNA genes, and operon B contains a second copy of the 5S rRNA gene. Using genomic DNA (gDNA) as template, the 48 newly constructed strains were tested with a forward primer that was designed to bind in the conserved region upstream of the 16S rDNA and a reverse primer binding within the transposon (Fig. 2b and Supplementary Fig. S1; primers AD53 and 54). The gDNA from *P. putida* pig-r1, pig-r2 and wildtype strain KT2440 were used as positive and negative controls, respectively. As expected, PCR with the wildtype gDNA gave no product, surprisingly however, PCR bands were obtained for all strains carrying the transposon (Supplementary Fig. S2), indicating that the *pig* genes were inserted into the rDNA in all 50 pig-r strains. Furthermore, the detection of different PCR fragment sizes was a first indication for varying distances of the *pig* gene insertion sites relative to the primer AD54 binding site located within the promoter regions of all *rrn* operons.

To verify this and further specify exact insertion loci that were previously also unknown for *P. putida* pig-r1 and pig-r2, we performed a detailed PCR analysis of all pig-r strains. To this end, seven primers (AD106-112) that specifically bind to the individual upstream regions of the respective *rrn* operons were used together with the TREX primer AD53 for generating PCR products in strict dependence on the respective TREX-*pig* insertion site (Supplementary Fig. S1). The operon *rrnB* represents a special case, as it is preceded by *rrnA*. Specific PCR bands were obtained for all strains so that insertion into distinct *rrn* operons could be assigned (Supplementary Fig. S3). The insertion sites were located in all of the seven *rrn* operons. However, the number of insertion events was not equally distributed: for example, only two strains carrying the TREX-*pig* transposon in *rrnF* could be identified, whereas eleven strains carried the recombinant transposon in *rrnC*. Estimation of PCR product sizes furthermore enabled mapping of each transposon insertion locus within the specific *rrn* operons and the exact positions were further verified by sequencing of PCR products (Fig. 2a). Figure 2b exemplarily depicts the strategy for the determination of transposon localization in *rrnC*, and a detailed summary of the methodology and results is given in Supplementary Fig. S1–S3 and Table S2.

In the majority of the tested strains, the insertion site was found to be within a 16S or a 23S rRNA structural gene, without a recognizable pattern therein. *P. putida* strain pig-r45 represents the only exception where the transposon inserted right before the start of the 16S rRNA structural gene as annotated in the ‘Pseudomonas Genome Database^27^’. None of the strains carried the *pig* genes in the region between the rRNA-coding sequences or in the tRNA genes. Interestingly, the fraction of intensely red clones we found after transposition and that were now confirmed to carry the *pig* genes in the rDNA (0.1%) is in a similar range as the proportion of 16S and 23S rDNA in the genome of *P. putida* KT2440 (0.25%; the *rrn* operons A, C and D, which were overrepresented as insertion loci in our sample set and were associated with strongest production, accounting for 0.1% of the genome) (see Supplementary Table S3 for a summary). The 16S and 23S rRNA-coding sequences therefore appear generally promising for integration and expression of a biosynthetic gene cluster.

### Correlation of *pig* gene insertion loci and prodigiosin production

**Figure 3:**
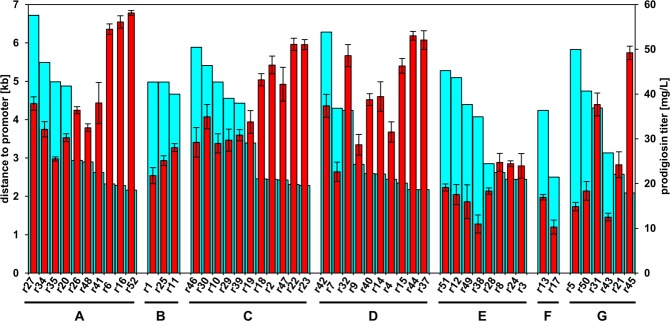
Correlation of rDNA insertion loci and promoter distances with prodigiosin titers in *P. putida* pig-r1 to pig-r52. Turquoise bars indicate the distance of the translation start of *pigA* to the *rrn* promoter [left axis, kb]. Red bars represent prodigiosin titers [right axis, mg/L]. Results of individual strains are grouped by the *rrn* operons in which the *pig* genes were inserted (A–G). In addition, prodigiosin titers of strains harboring the *pig* genes within the same *rrn* operon were sorted by the promoter distance in descending order. Distances up to 3.4 kb (r19) indicate insertion into the 16S rRNA gene and above 4.1 kb (r38) insertion into the 23S rRNA gene (also see Fig. 2a). Prodigiosin titers are mean values of triplicate measurements with the respective standard deviation.

Individual prodigiosin production titers of the 50 *P. putida* strains carrying the *pig* genes in the rDNA were analyzed after cultivation in liquid medium to unravel putative correlations with their specific insertion loci. To this end, cells were grown at small scale in TB medium, which was previously determined to be suitable for high-level prodigiosin production^11^, and end-point product levels were determined after 24 hours based on prodigiosin absorption in ethanol extracts. Afterwards, product titers of the pig-r strains were assigned to the specific *rrn* operons where *pig* genes were inserted, and moreover sorted within the respective *rrn* operon group with respect to the individual distances of the -10 region of the promoters immediately upstream of the 16S rRNA genes to the translation start of the first gene of the *pig* gene cluster, i.e. *pigA* (Fig. 3). Note that the minimal possible distance between the start codon of *pigA* and the *rrn* promoter is 1.9 kb due to the position and length of the L-TREX cassette and the gene *cueR* between these two DNA elements (see Fig. 1a).

Based on the set of pig-r strains, several general trends could be observed: (i) Prodigiosin production reached maximal values of ~50–60 mg/L in strains with insertion loci of the *pig* genes in the *rrn* operons A, C, D and G, while strains with insertion loci in the *rrn* operons B, E and F only reached maximal titers of 16–28 mg/L. Evaluation of mean titers of those strains harboring the *pig* genes within the same *rrn* operon showed that strains carrying the *pig* genes in *rrn* operon A, C and D exhibited generally high prodigiosin accumulation (average ~40 mg/L). In contrast, *pig* gene insertion in *rrn* operon B and G resulted in intermediate production levels (average ~25 mg/L) whereas final product titers were even lower in strains with operon E and F as insertion sites (average ~20 mg/L and lower). (ii) Furthermore, in those strains carrying the *pig* genes within the same *rrn* operon, a correlation between promotor distance and prodigiosin production becomes apparent: here, a larger distance between the *pig* genes and the *rrn* promoter (max. ca. 6.6 kb) mostly correlated with lower production levels whereas shorter distances (min. ca. 2 kb) typically resulted in higher production levels, most clearly for *rrn* operons A, C and D which are best represented in the data set. This trend seemed especially pronounced in *P. putida* strains where *pig* genes were located within in the 16S rRNA gene (see also Supplementary Fig. S4). Interestingly, the strains pig-r33 and pig-r36, which were excluded from our main study due to substantially weaker coloration than the other pig-r strains, accordingly showed a much lower production (below 0.1 mg/L prodigiosin), corroborating our visual assessment, and were tested negative for insertion in the rDNA by the PCR screen.

Since strains with *pig* gene insertion in some *rrn* operons were underrepresented in the initially generated set of intense red strains, we additionally created another TREX-*pig* library comprising ca. 30,000 clones that were specifically tested for gene insertion within the *rrn* operons B, E, F and G. To this end, we now pre-selected clones with a wider range of colony color phenotypes from light pink to intense red because our above described results (Fig. 3) pointed to lower prodigiosin production by strains with transposon insertion in the underrepresented operons. With these loosened screening criteria, we found a higher fraction of clones within the library to exhibit the target phenotype (1%) than in the initial screening where only very red clones were accepted (0.1%). However, among those exhibiting the target phenotype, the proportion of clones carrying the transposon in the rDNA was significantly lower than in the initial set: out of more than 300 clones with the target phenotype, a random sample of 132 clones was subjected to PCR analysis using the respective primer pairs to indicate the integration into rDNA generally, and into *rrn* operons B, E, F and G specifically. We found 59 clones to carry the TREX-*pig* transposon in the rDNA and identified another set of 19 strains with insertion loci in the targeted operons (Supplementary Table S3). Investigation of insertion sites, that were assigned to individual *rrn* operons on the basis of PCR analysis as described above, and prodigiosin titers corroborated hitherto described trends. Due to the different screening procedure to the main data set (Fig. 3), the results are shown separately (Supplementary Fig. S5).

Our findings thus point to the rDNA as a uniquely suitable chromosomal insertion site for *pig* gene expression implementing a heterologous prodigiosin pathway and, here, to an influence on production performance depending (i) on the *rrn* operon, in which *pig* genes were inserted, and, in addition, (ii) on the distance to the respective *rrn* promoters. The latter finding is remarkable considering that the total length of the *pig* gene cluster including *cueR* is 21.8 kb and a comparably minor difference in promoter distance of few kb or less had a major impact on production levels.

### Transcript levels of *pig* genes inserted into different rDNA loci

**Figure 4:**
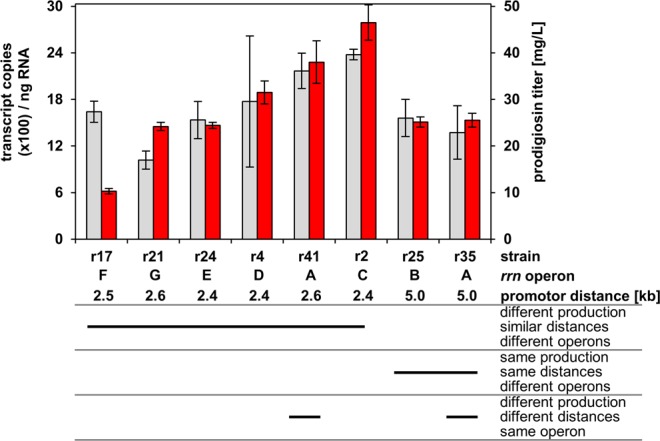
Correlation of individual rDNA insertion loci with *pig* transcript levels in *P. putida*. Grey bars indicate transcript levels of *pigN*, the last gene in the *pig* cluster as an indicator of complete *pig* gene transcription [left axis, 100 transcript copies/ng RNA]. Red bars represent prodigiosin titers [right axis, mg/L] as also shown in Fig. 3. Names of *P. putida* strains and TREX-*pig* insertion loci specifications, i.e. *rrn* operons (A–G) and distances between the *rrn* promoter and *pigA* start, are indicated. Based on the criteria prodigiosin production, distance of *pig* genes to the *rrn* promoter, and insert location in a particular *rrn* operon, strains were grouped as indicated below the diagram. Prodigiosin titers and transcript copy numbers are mean values of triplicate measurements with the respective standard deviation.

To assess if different prodigiosin production titers of *P. putida* strains with *pig* gene insertion at different positions in the rDNA were correlated with differential *pig* gene transcript levels, we exemplarily investigated *pig* gene mRNA in selected strains. To this end, the transcript of the last gene of the *pig* gene cluster, *pigN*, which is most distal to the promoter, was quantified in *P. putida* strains during cultivation in TB medium by RT-qPCR as an indicator of complete transcription of the entire *pig* gene cluster (Fig. 4). We first examined the influence of insertion into different *rrn* operons on *pig* gene mRNA levels. Therefore, strains carrying *pig* genes in similar short distances to the promoters (2.4–2.6 kb) of the *rrn* operons A, C, D, E, F, and G were tested. In addition, two strains with more distal insertions (5.0 kb) in operons A and B were analyzed. Within this data set, a good correlation of *pig* gene transcript levels and product formation was found (Supplementary Fig. S6), corroborating the role of the individual *rrn* operons and their promoters for transcription efficiency and thus on final prodigiosin production, except for strain pig-r17, which had a relatively low prodigiosin titer and strain pig-r21, which had a relatively high product titer. Within *rrnA*, the longer distance to the *rrn* promoter yielded lower transcript levels, possibly due to increased RNA polymerase drop-off or decreased stability of the mRNA 3′ terminus.

Our results indicate that prodigiosin production appears to be generally correlated to *pig* gene transcript levels and both, the specific properties of the individual *rrn* operon as well as the distance between the promoter and the *pig* gene cluster seem to be key factors determining the level of pathway gene expression in *P. putida* KT2440.

The expression of bacterial *rrn* operons is generally thought to be correlated with growth to provide suitable ribosome concentrations matching cellular protein metabolism^28^ and moreover differentially influenced by environmental factors^29,30^. Recently, the medium composition has been shown to strongly affect the expression of different *rrn* operons in *E. coli*^30^, with TB rich medium and M9 minimal medium leading to differential expression patterns. For an initial assessment if *rrn* expression in *P. putida* might be influenced in a similar way by nutrient limitation, we also tested the minimal medium for comparison of prodigiosin production by selected strains in rich TB vs. minimal M9 medium. Interestingly, product levels were significantly reduced in M9 compared to cultivation in TB medium, but the production pattern was conserved: strains showed the same tendency to group as low-level producers (pig-r17), intermediate producers (pig-r21, -r25, -r35) and high-level producers (pig-r41, -r2) in both media (Supplementary Fig. S7), possibly pointing to intrinsic factors as major determinants of differential *rrn* operon expression.

**Figure 5:**
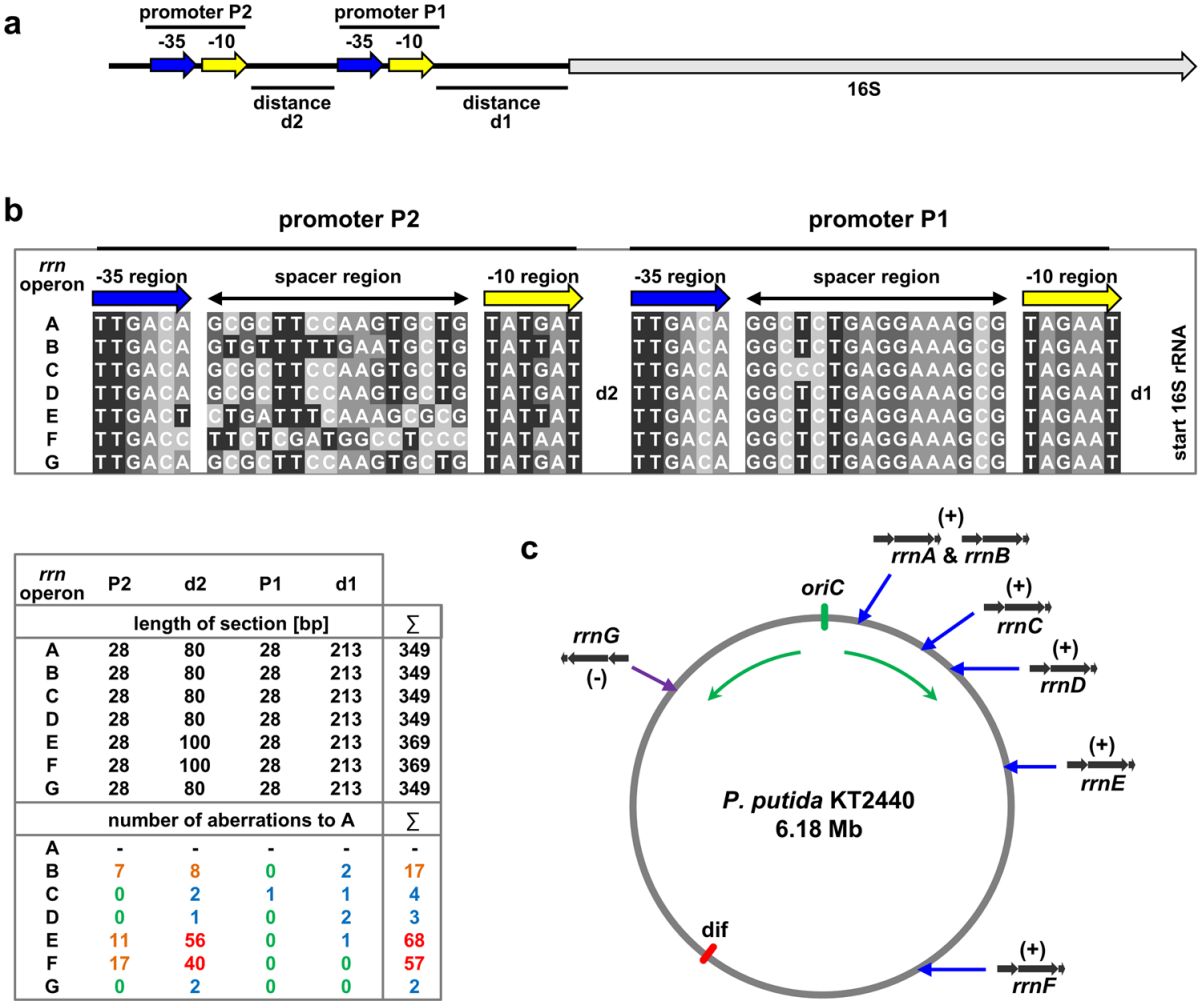
Promoter sequences and chromosomal locations of *P. putida* KT2440 *rrn* operons. (**a**) General structure of upstream regions of the rRNA-encoding *rrn* operons with the two proposed promoter sequences P1 and P2 (not drawn to scale). (**b**) Sequence comparison of the seven promoter regions of *rrn* operons A-G. The sequences of the two promoters as well as the position of the distance regions located between the 16S rRNA structural gene and promoter P1 (d1), and between the two proposed promotor sequences (d2) are shown. The length of each section and the respective number of nucleotide aberrations to the promoter sequence of *rrnA* in form of different nucleotides, gaps or insertions are listed. Color scheme indicates no (0, green), minor (1–4, blue), moderate (5–17, orange) and substantial (>18, red) aberrations. The complete sequence alignment is shown in Supplementary Fig S7. (**c**) Schematic of the *P. putida* chromosome and the locations of the *rrn* operons on the (+) or (−) strand. The positions of the proposed chromosomal origin of replication (*oriC*), the direction, and the terminus of replication (*dif* ) are indicated.

The role of the individual *rrn* operons in determining *pig* gene transcript levels may be due to (i) differential strength of the seven *rrn* operon promoters and (ii) distinct effects of their positions in the bacterial chromosome. We therefore further analyzed the upstream sequences of the seven *rrn* operons of *P. putida* KT2440 in a comparative alignment to identify specific differences in their promoter sequences (the complete alignment is shown in Supplementary Fig. S8). Although the promoters of the *rrn* operons have not been characterized specifically for *P. putida* KT2440 in detail, their positions can be deduced because of their universal nature from sequence similarities to other bacteria and the typical consensus motifs: all upstream regions comprise two potential promoter sequences exhibiting typical -10 and -35 regions of sigma-70 RNA polymerase-dependent promoters as highlighted in yellow and blue, with the one predicted promoter sequence that is located directly upstream of the 16S rRNA gene here denoted as promoter P1 and another one further upstream of the first promoter designated as promoter P2 (Fig. 5a).

Since the exact sequence of the conserved -10 and the -35 regions as well as the spacer region between them are known determinants of promoter activity^8,9^, the specific differences of these promoter sequences were investigated, using the sequence belonging to *rrnA* as a reference. In addition, the sequence of the distance regions between the promoter P1 and the 16S rRNA gene, and the sequence between both promoters, here designated as d1 and d2, respectively, were compared (Fig. 5b). The two -10 and -35 regions as well as the spacer region between them are identical for two of the *rrn* operons (A and D), in which *pig* gene insertion led to highest production titers (see Fig. 3). The respective sequences of the other *rrn* operons exhibited different numbers of deviations from the reference, primarily within the sequence of promoter P2. While the promoters of *rrnC*, which likewise enabled high prodigiosin production titers upon insertion of *pig* genes, only contain one different nucleotide in a spacer region, those of operons E and F, which were associated with lower product titers, show multiple aberrations not only in the spacer region but also in the -10 and -35 regions. For the same operons, a distance d2 between the two promoter sequences different from all other cases was additionally noted. In contrast to the d1 sequence, where no or only minor aberrations were observed, the d2 sequence moreover harbors moderate (*rrnB*) and substantial (*rrnE* and *rrnF*) aberrations. The overall number of sequence aberrations roughly correlated with production tendencies, i.e. mean and maximal titers, for the six *rrn* operons A, B, C, D, E and F. However, the promoters and distance sequences d1/d2 of *rrnG*, which was associated with intermediate production upon *pig* gene insertion, exhibit only a minor number of total aberrations from the respective sequences of *rrnA*. In conclusion, the differences in promoter sequences of different *rrn* operons in the here investigated set of strains carrying the TREX-*pig* transposon may significantly contribute to the differential transcript and prodigiosin production levels we observed. However, these differences alone cannot account for the observation that some *rrn* operons appear to be more favorable insertion sites for production than others.

The different genomic contexts of the *rrn* operons of *P. putida* KT2440 are likely additional determinants for the efficiency of heterologous gene expression, prompting further inspection. First, the *rrn* operons are located in individual genomic regions containing different types of upstream sequences in front of their promoters and their expression levels can be affected by various factors including upstream activating sequences, terminators and read-through effects. While their interplay has not been studied for the *rrn* operons and is difficult to predict, it is conspicuous that genes and consequently the respective promoters upstream of the *rrn* operons A, C and D, where insertion led to highest production titers, are oriented in the same direction as the rRNA-encoding genes (see Fig. 2a). This might account for potential additional transcriptional read-through – even though ARNold-predicted rho-independent transcription terminators^31^ occur behind the preceding genes of *rrn* operons C and D^32^.

Further, the *rrn* operons are located at different positions across the chromosome of *P. putida* KT2440. Since the genomic region in proximity to the origin of chromosomal replication *oriC* is generally associated with a higher gene dosage^33^ which may be correlated with gene expression^34^, we inspected the positions of the *rrn* operons in relation to the *oriC* (Fig. 5c). Although the *oriC* and the corresponding termination *dif* of *P. putida* KT2440 have not been characterized in detail, their positions were deduced from sequence similarities to other bacteria^35–38^. While the *rrn* operons A, B, C and D are relatively close to the predicted *oriC*, the *rrn* operons E, G and especially F are located at a considerable distance from *oriC*. The *rrn* operons F and G are, however, in a similar distance to the terminus of replication, since the proposed *oriC* and *dif* regions partition the chromosome in two asymmetrical replicores. With the exception of *rrnB*, this distribution largely corresponds to the here described production capabilities of strains carrying biosynthetic genes in the *rrn* operons and might corroborate gene dosage effects. Notably, *rrn* operons A, B, C, D, E and F are all located in the same replicore, while G is the only one in the other replicore, however, their positions on the (+) and (−) strands, respectively, account for an orientation in the direction of replication in all cases, so that adverse collision effects can be excluded.

### Long-term stability of prodigiosin production in *P. putida* TREX-*pig* strains

**Figure 6:**
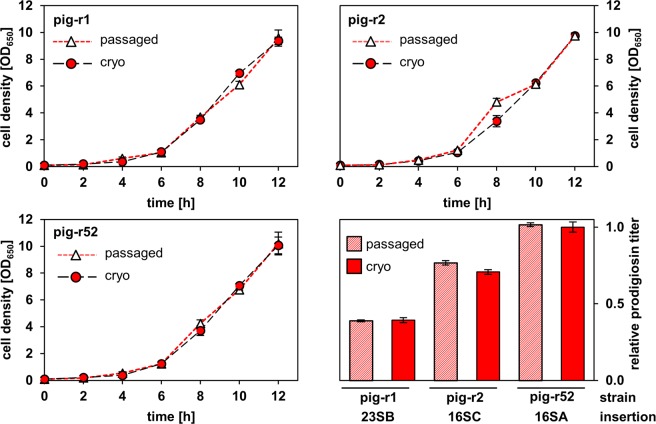
Long-term stability of growth and the recombinant prodigiosin pathway in *P. putida* pig-r1, pig-r2 and pig-r52. For each strain, cell growth and relative prodigiosin titers of freshly re-cultivated cells, which were stored in cryo-conservation over 2 years (red), were compared to growth and product titers of cells, which were repeatedly passaged on agar plates over 2 years (red/white). Prodigiosin titers were normalized in relation to the production of the strongest producer strain *P. putida* pig-r52 (cryo). Cell densities and prodigiosin titers are mean values of triplicate measurements with the respective standard deviation.

The maintenance of a stable chromosomal integration and the expression performance of biosynthetic genes represents a major challenge in natural products biotechnology^7^. In general, rDNA appears to be a stably conserved locus^39,40^. In order to assess the general applicability of *P. putida* rDNA sites as integration loci in terms of long-term genetic stability and natural product formation, the following strains were subjected to a stability test: (i) *P. putida* pig-r1 which showed lower production titers and carried the *pig* genes in the 23S rRNA gene of *rrnB*, (ii) pig-r2 (intermediate prodigiosin production, insertion in the 16S rRNA gene of *rrnC*) and (iii) pig-r52 (high prodigiosin production, insertion in the 16S rRNA gene of *rrnA*) (see Fig. 2a and Fig. 3). To evaluate the long-term stability of these stains, the cell growth and prodigiosin production titers were analyzed after 24 plate-to-plate passages of single colonies over 2 years in direct comparison with strains, which were preserved in cryo-conservation at −80 °C (Fig. 6).

Remarkably, growth and prodigiosin production of the tested strains were basically the same after repeated passaging as compared to cryo-conserved cells. Only minor differences in growth were observed during the exponential phase, but all cell cultures reached the same cell density of ca. 10 (OD_650_) after 12 h. This corroborates the remarkable resistance of the host to the product prodigiosin, which acts as an antibiotic against other bacteria, and, more importantly, points to the genetic stability and robust expression of clustered genes that are localized within the *P. putida* rDNA.

## Discussion

In this study, we have described the rRNA-encoding *rrn* operons of *P. putida* KT2440 as exceptionally suitable sites to be “hijacked” for recombinant gene cluster insertion and expression. This was demonstrated by (i) selection of prodigiosin producing strains after random chromosomal integration of the *pig* gene cluster, (ii) substantial but *rrn* operon-dependent differential expression and prodigiosin production, as well as (iii) a remarkable long-term stability of the TREX-*pig* containing strains. Our observations indicate that the chromosomal rDNA sites appear to exhibit unique characteristics, which are favorable for effective gene cluster insertion and expression.

A substantial and concerted transcription of biosynthetic genes is necessary to implement a foreign biosynthetic pathway within a heterologous production host. In our study, formation of the desired end product clearly demonstrates that *pig* gene insertion into the rDNA led to the full transcription of the 21 kb gene cluster, which was confirmed by RT-qPCR analysis of the last gene in the *pig* cluster. The *rrn* operons are generally considered to be among the most highly expressed genes in bacteria^16^. This suggests that biosynthetic genes inserted here can be transcribed at a substantial level as well. Linking heterologous gene cluster transcription to rDNA activation may also grant most efficient use of cellular resources as the transcription of the recombinant biosynthetic genes will occur when the cell is also providing the machinery needed for the protein production. Notably, full transcription in this fashion can only be achieved if the employed gene cluster consists of unidirectionally organized genes with no transcription termination signals in between. In microorganisms, genes coding for large modular biosynthetic pathways are often, like the *pig* cluster, unidirectionally organized. However, with techniques like recombinational cloning^12^, gene clusters can otherwise be rearranged to a unidirectional structure. This might, however, compromise a required balance in transcript levels of individual genes and needs to be tested experimentally. Alternative approaches include systematic gene cluster and promoter refactoring which can serve to install beneficial transcription strengths^41,42^.

We further noted substantial differences of transcription and hence prodigiosin production in dependence of the specific *rrn* operon carrying the biosynthetic genes, in that the *rrn* operons A, C and D accounted for highest, B and G for intermediate and E and F for lowest levels, which was largely in concordance with differential promoter sequences. The individual expression levels of the different *rrn* operons cannot be easily quantified in transcriptome studies due to their high sequence identity so that respective information on the *P. putida rrn* genes was previously unavailable. Our present study thus provides an initial characterization of cellular processes that hitherto remained a blind spot. For diverse other bacteria including *Escherichia coli*^43^ or *Lactobacillus plantarum*^44^, varying strength of the different *rrn* promoters has been reported. Also for Pseudomonads, a differential promoter strength has been indicated^29^. In addition, a gene dosage effect at locations near the origin of chromosomal replication can be assumed. For *E. coli*, it was shown that expression levels of genes which are under control of a constitutive promoter correlate with the distance of the genes to the *oriC*, as more copies of the target genes are available for transcription when they are close to the origin of chromosome replication^16,34^. This effect can be substantial under the conditions tested, since the majority of cells in a fast-growing *P. putida* culture have more than two chromosomal copies^45^. Finally, a co-localization effect of the target biosynthetic genes with the transcription machinery is thinkable: it is known that the transcription and translation machinery represent limited resources in bacterial cells which are subject to competitive interaction by different modules of the cellular circuitry^46^. Moreover, in *P. putida*, transcriptional and translational machinery are located in spatially distinct positions in the cell^47^ and it has been discussed, at least for *E. coli*, that *rrn* operons substantially recruit the transcription machinery thus forming transcription foci with high RNA polymerase availability^48^. Therefore, the transcription level of genes integrated in the *rrn* operons A–D, which occur in relative proximity to one another, may be especially positively influenced by a local focus of the transcription machinery that is generated here. The thus observed differential expression of the reporter gene cluster inserted into the *rrn* operons A–G, which led to production differing by a factor of six, may be exploited to implement the optimal expression level for a given biosynthetic pathway.

Overall, a close proximity to the *rrn* promoter seems to be favorable for higher expression and production levels according to our findings. Assuming that the transcriptional activity for each *rrn* promoter is not affected by the insertion of the *pig* construct, the variability in prodigiosin production observed for strains with different insertion loci within one *rrn* operon could additionally be the result of structural effects on mRNA level. The transcription of the heterologous gene cluster in an rDNA background generally results in an rRNA-mRNA hybrid, where the *pig* mRNA is flanked by upstream and downstream regions of rRNA fragments. Usually, rRNA remains untranslated and forms secondary structures which are part of the ribosome^49,50^. Due to these structures, rRNA, unlike mRNA, is exceptionally stable and only degraded under specific conditions^51^. In an rRNA-mRNA hybrid, extensive base pairing of the flanking rRNA regions thus may co-stabilize the transcript by protecting the mRNA from nuclease activity^52^. Loop structures formed by the flanking rRNA segments can further improve the accessibility of motifs necessary for efficient translation, e.g. the RBS for proper ribosome recruitment, and enable higher translation rates^53^. Clones carrying an insertion in the spacer region between the 16S and 23S rRNA genes were not identified. This region is subject to cleavage by several RNases during the process of rRNA maturation^49^, possibly leading to instable and untranslatable RNA-hybrids. However, interactions between rRNA and mRNA might also have a negative impact on translation rates by blocking sites essential for effective translation. These assumptions are underlined by several outliers, such as strains pig-r32 (Fig. 3) and -r17 (Fig. 4), for which the distance to the promoter clearly is not the only determinant for their production profiles. While promoter proximity is thus certainly a major determinant, local effects can potentially also heavily influence transcript stability and translation efficiency. In future studies, the targeted insertion of gene constructs at the same integration site in the different *rrn* operons could normalize for these effects, enabling a direct comparison of the transcriptional output of each operon. For the development of a production process, it would be highly interesting to closely study the dynamics of promoter activity, mRNA stability, functional enzyme formation and biosynthetic conversions, and to determine most beneficial constellations. In addition, a systematic assessment with different gene clusters may reveal length- or sequence-specific effects and limitations.

Stable maintenance of biosynthetic DNA is a prerequisite for effective long-term production and a central issue in production strain development. We have shown remarkable stability of prodigiosin producing strains which carry the *pig* genes in the rDNA. Potentially, the rDNA poses an especially favorable chromosomal site in this regard. Microorganisms seem to have evolved strategies to specifically ascertain correct replication of this essential feature. In bacteria, certain proteins were shown to preferentially associate with the *rrn* operons in a transcription-dependent manner to cooperatively reduce replication-transcription conflicts at these highly transcribed genes^39,54^. One might speculate that protective mechanisms specifically directed toward the rDNA might also protect the integrated recombinant genes from accumulating errors during replication. Such phenomena might explain the here observed remarkable stability of prodigiosin producing strains after many generations. However, this idea was not specifically addressed in our study and no control strains with the *pig* genes inserted in another chromosomal site were subjected to the long-term investigation for comparison. Further studies are necessary to provide evidence on the matter.

Although the discussed unique features recommend the rDNA as highly attractive chromosomal site for gene cluster insertion and expression, disturbing such a vital element of the genome might be expected to exert adverse effects on cellular fitness and production capacities. Indeed, the presence of multiple copies indicates a fundamental importance of *rrn* genes for bacterial fitness, as shown *via* artificial reduction of the number of *rrn* operons in *Bacillus*^15,55^. However, we did not observe any apparent fitness defects in our set of strains in the present study and showed in a previous study that growth of *P. putida* strain pig-r2 in shaking flasks was comparable to that of the wildtype *P. putida* KT2440^11^. In addition, other *P. putida* strains such as S12 or S16 only have six *rrn* operons^56,57^ with little noticeable effect on the maximum growth rate under laboratory conditions^58^, indicating a certain plasticity in this trait for the *P. putida* clade. Likewise, it has been reported elsewhere that the deletion of a single *rrn* operon of *Bacillus subtilis* and *E. coli* does not lead to apparent fitness defects^15,59^. An explanation for these findings might be that the requirement of a certain number of *rrn* operons seems to be context-dependent. Specifically, it was suggested that the redundancy of the *rrn* operon plays a role in the adaptation to different environmental conditions^60–62^ and bacteria with more *rrn* operon copies are considered more adaptable to changing nutrient availability^63–65^. Under constant laboratory conditions, bacteria appear to compensate the loss of one or two *rrn* operons by upregulation of others to maintain the required amount of rRNA, as was shown for *E. coli*^66,67^.

In summary, our study demonstrates the usability of a natural key feature of the bacterial genome for production strain construction. In the future, expression of biosynthetic pathways in an rDNA context of amenable hosts like *P. putida* may be used to generate production strains for various natural products. This shall contribute to establishing high-level stable production strains of known compounds, and to the exploitation of yet unknown pathways or silent gene clusters.

## Methods

### Bacterial strains and culture conditions

*Escherichia coli* strain S17-1^68^, which was applied for conjugation, was cultivated at 37 °C under constant agitation (120 rpm) in shake flasks in liquid LB medium (by Carl Roth^®^, Karlsruhe, Germany; composed of 10 g/L tryptone, 5 g/L yeast extract, 10 g/L sodium chloride) or on LB agar plates (LB medium completed with 15 g/L Agar-Agar, Kobe I by Carl Roth^®^). *Pseudomonas putida* strain KT2440^13^ was cultivated, unless stated otherwise, at 30 °C under constant agitation (120 rpm) in shake flasks in LB liquid medium or on LB agar plates. Antibiotics were supplemented, where appropriate, to the following final concentrations: 10 µg/mL tetracycline (*E. coli*); 25 µg/mL gentamicin, 25 µg/mL irgasan (*P. putida*).

### Generation of *P. putida* prodigiosin production strains

Following previously established protocols^10–12^, vector pTREX-pig^10^, which carries the *pig* gene cluster from *Serratia marcescens* W838, was transferred to *P. putida* KT2440 *via* conjugation using *E. coli* S17-1 as donor. Since the origin of replication in pTREX-pig is pMB1, replication is not supported in *P. putida*. Therefore, positive selection of *P. putida* clones from the conjugation mix, in which Tn5 transposition of the recombinant TREX-*pig* transposon comprising the entire prodigiosin biosynthetic *pig* gene cluster occurred, could be conducted as previously described^10,11^ by using LB agar plates supplemented with gentamicin because the transposon includes a respective resistance gene. In addition, irgasan was added to selectively allow *P. putida* growth but prevent *E. coli* S17-1 growth. Identification of prodigiosin production strains among exconjugants was facilitated by their red colored colony phenotypes on agar plates. Thusly selected clones were streaked on agar plates in distinct patterns to enable a visual comparison of coloration.

### Production and quantification of prodigiosin

Prodigiosin production was conducted essentially as previously described^11^. Prodigiosin producing *P. putida* strains (*P. putida* pig-r1 to pig-r52) were grown as precultures in liquid TB medium (by Carl-Roth Karlsruhe, Germany ‘Terrific broth, modified’; composed of 12 g/L Casein, enzymatically digested, 24 g/L yeast extract, 9.4 g/L dipotassium phosphate, 2.2 g/L monopotassium phosphate, 4 mL/L glycerol) or in M9 minimal medium (composed of ‘M9 Minimal Salts’ by Sigma-Aldrich, supplemented with 0.2% glucose, 1 mM MgSO_4_, 0.1 mM CaCl_2_, as described by Kurylo *et al*.^30^). Production cultures were inoculated from precultures in FlowerPlates^®^ (by m2p-labs GmbH, Baesweiler, Germany) to a starting cell density of OD_650_ = 0.05 in 800 µL of TB or M9 medium per well. A ThermoMixer^®^ C (by Eppendorf AG, Hamburg, Germany) or a TiMix MTP-shaker with a TH Incubationhood (by EB GmbH, Hechingen, Germany) was used for incubation of production cultures at 20 °C under constant agitation (1400 rpm). Samples with cell masses equivalent to 1 mL cell suspension with a density of OD_650_ = 0.3–1 (GENESYS™ Spectrophotometer, ThermoFisher Scientific GmbH, Waltham, USA), equivalent to ca. 0.2-0.6 mg dry cell weight, were harvested after 24 h of cultivation by centrifugation. Cells were extracted with 1 mL ethanol (4% of 1 M HCl), cell debris was removed by centrifugation and absorbance spectra from 450 to 600 nm were recorded to verify prodigiosin-specific spectra with the typical maximum at 535 nm. Prodigiosin content in the extracts was determined *via* the molar extinction coefficient (ε_535_ [M^−1^ cm^−1^] = 139,800) in the solvent as described before^11,12^ and calculated as prodigiosin titer in mg per liter culture.

### Determination of chromosomal integration loci of the TREX-*pig* transposon within the rDNA

For the determination of the chromosomal integration loci of the TREX-*pig* transposon in all 50 prodigiosin production strains, a PCR-based method was employed, using Phusion^®^ High-Fidelity DNA-Polymerase (ThermoFisher Scientific GmbH, Waltham, USA) for DNA amplification in a Tprofessional Basis Gradient Thermocycler (Biometra GmbH, Göttingen, Germany). Genomic template DNA was isolated with the DNeasy Blood & Tissue Kit (Quiagen^®^ GmbH, Hilden, Germany). In a first step, integration into any rDNA region was detected by PCR amplification using oligonucleotide AD53, which binds within the L-TREX cassette of the TREX-*pig* transposon, in combination with oligonucleotide AD54, which binds within the highly conserved upstream regions of every *rrn* operon and subsequent analysis of PCR products by gel electrophoresis. Genomic DNA was subjected to another PCR to determine the specific *rrn* operon as well as the accurate integration loci within the genome of *P. putida* KT2440. Therefore, oligonucleotide AD53 in combination with a specific oligonucleotide for each *rrn* operon (*rrn* operon A: AD106, B: AD107, C: AD108, D: AD109, E: AD110, F: AD111, G: AD112) were employed. PCR products were analyzed with agarose gel electrophoresis, isolated using innuPREP DOUBLEpure Kit (Analytik Jena AG, Jena, Germany) and sequenced using oligonucleotide AD134 and AD63. Sequencing results were analyzed based on the Pseudomonas Genome DB^27^ to verify the assigned *rrn* operon and to determine the exact integration loci of the TREX-*pig* transposon. Commercial services were engaged for DNA synthesis of primer oligonucleotides and for DNA sequencing (Eurofins Genomics GmbH, Ebersberg, Germany). Described PCR primer and sequencing primer binding sites are shown in Supplementary Fig. S1 and Fig. 2, respectively. All used oligonucleotides are listed in Supplementary Table S3.

### RT-qPCR analysis of transcript levels

Cells were harvested from production cultures during the exponential growth phase (8 h after inoculation, at cell densities of OD_650_ = 1.5. RNA was extracted from cell material corresponding to OD_650_ = 1.5 using the NucleoSpin® RNA Kit (Macherey-Nagel). During the procedure, 80 μL DNaseI-Mix (10 μL DNaseI Stock + 70 μL RDD buffer) of the RNase-Free DNase Set (Quiagen) was applied onto the NucleoSpin® RNA column for a 15 min incubation. Total RNA was eluted with 47 μL nuclease-free water and additionally treated with the DNA-free™ DNA Removal Kit (Life Technologies). Reverse transcription was conducted in 20 µL samples using the Maxima First Strand cDNA Synthesis Kit for RT-qPCR (ThermoFisher) with random hexamer primers and 1000 ng RNA as template. After dilution of the samples with water to generate a total volume of 92 µL, 9.2 µL were subjected to qPCR in a reaction mix with 10 µL Maxima SYBR Green/ROX qPCR Master Mix (2×) and 0.8 μL primer mix for the detection of *pigN* or *rpoD* transcripts (10 pmol per oligonucleotide), respectively, in the 7900HT Fast Real-Time PCR System running 30 PCR cycles. The primers were designed using the Primer3Web suite^69^. For calibration, the plasmid pPIG^10^ was used as template (in concentrations ranging from 1 × 10^−3^ to 1000 ng), allowing calculation of transcript copy numbers *via* the molecular weight of detected DNA fragments. Signals of *rpoD* transcripts were additionally detected as internal reference to monitor RNA extraction and cDNA generation efficiency, and employed for correction of *pigN* signals by using the deviation of the internal reference signal from the average internal reference signal as a correction factor.

### DNA sequence analyses

All *P. putida* KT2440 genome annotations, sequences and locus tags used for sequence alignments or general analysis were obtained from Pseudomonas Genome DB (http://www.pseudomonas.com)^27^ using the most recent update (AE015451.2)^14^ of the *P. putida* KT2440 genome sequence (AE015451) first published by Nelson *et al*.^13^. For multiple sequence alignments, Clustal omega (http://www.ebi.ac.uk/Tools/msa/clustalo/)^70^ was utilized, for pairwise and global alignments, BLAST (http://blast.ncbi.nlm.nih.gov/)^71^ was employed. Consensus promotor sequences of the seven *rrn* operons were predicted with the help of the BPROM analysis tool (http://www.softberry.com/berry.phtml)^72^; rho-independent terminator sequences were predicted using ARNold (http://rna.igmors.u-psud.fr/toolbox/arnold/)^31^.

### Ethical approval

This article does not contain any studies with human participants or animals performed by any of the authors.

## Supplementary information

Supplementary Information

## Data Availability

All data generated or analyzed during this study are included in this published article (and its Supplementary Information files).
