# Supplementary material for: Pseudomonas putida rDNA is a favored site for the expression of biosynthetic genes
Source: Sci Rep. 2019 May 7;9:7028. doi: 10.1038/s41598-019-43405-1 (PMC6505042; doi:10.1038/s41598-019-43405-1)
Supplement: Supplementary file 1 — Supplementary Information [file 41598_2019_43405_MOESM1_ESM.pdf]

SUPPLEMENTARY INFORMATION

Pseudomonas putida rDNA is a favored site for the expression of biosynthetic genes

Andreas Domröse[1], Jennifer Hage-Hülsmann[1,2], Stephan Thies[1,3], Robin Weihmann[1,3], Luzie Kruse[1], Maike Otto[4], Nick Wierckx[4,5], Karl-Erich Jaeger[1,3,5], Thomas Drepper[1,3,*], Anita Loeschcke[1,3,*]

1, Institute of Molecular Enzyme Technology, Heinrich Heine University Düsseldorf, Forschungszentrum Jülich, Jülich, D-52425, Germany
2, Cluster of Excellence on Plant Sciences (CEPLAS), Heinrich Heine University Düsseldorf, Düsseldorf, D-40225, Germany
3, Bioeconomy Science Center (BioSC), Forschungszentrum Jülich, Jülich, D-52425, Germany
4, Institute of Applied Microbiology-iAMB, Aachen Biology and Biotechnology-ABBt, RWTH Aachen University, Aachen, D-52074, Germany
5, Institute of Bio- and Geosciences (IBG-1), Forschungszentrum Jülich, Jülich, D-52425, Germany
* corresponding authors:
Anita Loeschcke, a.loeschcke@fz-juelich.de
Thomas Drepper, t.drepper@fz-juelich.de

Content

Content	page
**Table S1. Identity matrices of the seven 16S and 23S rRNA genes of *P. putida* KT2440.**	**2**
**Figure S1. Primer binding sites for PCR screens of TREX-*pig* transposon insertion in rDNA of *P. putida* strains pig-r1 to pig-r52.**	**3**
**Figure S2. Results of PCR analysis for detection of TREX-*pig* transposon insertion in rDNA of *P. putida* strains pig-r1 to pig-r52.**	**4**
**Figure S3. Summary of PCR results for detection of TREX-*pig* transposon insertion in specific *rrn* operons of *P. putida* strains pig-r1 to pig-52.**	**5**
**Table S2. Chromosomal insertion loci in prodigiosin producing strains *P. putida* pig-r1 to pig-r52.**	**6**
**Table S3: Summary of *P. putida* TREX-*pig* libraries, selection procedures and conducted examinations.**	**7**
**Figure S4. Correlation of distances from *rrn* promoters to *pig* genes with prodigiosin production in *P. putida* pig-r1 to pig-r52.**	**8**
**Figure S5. Correlation of rDNA insertion loci and promoter distances with prodigiosin titers in a second library of *P. putida* strains carrying the TREX-*pig* transposon.**	**9**
**Figure S6. Correlation of *pig* transcript levels to prodigiosin production in *P. putida* pig-r strains.**	**10**
**Figure S7. Comparison of prodigiosin production and *pig* gene expression in TB and M9 medium.**	**11**
**Figure S8. Sequence alignment of promoter regions upstream of the seven *rrn* operons of *P. putida* KT2440.**	**12**
**Table S4. Oligonucleotides used in this study.**	**13**

Table S1. Identity matrices of the seven 16S and 23S rRNA genes of *P. putida* KT2440. Pairwise sequence comparison in ClustalOmega alignments was employed to determine percentage identities of rDNA sequences and the total number of sequence aberrations. The number of sequence aberrations is color coded with 0 in green, 1-2 in blue, 3-4 in orange, >4 in red.

16S	A	B	C	D	E	F	G	*rrn* operon
identity	100.00%	100.00%	99.93%	99.80%	99.93%	99.87%	99.87%	A
no. aberr.	0	0	1	3	1	2	2	
	identity	100.00%	99.93%	99.80%	99.93%	99.87%	99.87%	B
	no. aberr.	0	1	3	1	2	2	
		identity	100.00%	99.87%	100.00%	99.93%	99.93%	C
		no. aberr.	0	2	0	1	1	
			identity	100.00%	99.87%	99.93%	99.93%	D
			no. aberr.	0	2	1	1	
				identity	100.00%	99.93%	99.93%	E
				no. aberr.	0	1	1	
					identity	100.00%	100.00%	F
					no. aberr.	0	0	
						identity	100.00%	G
						no. aberr.	0	

23S	A	B	C	D	E	F	G	*rrn* operon
identity	100.00%	99.97%	99.90%	99.93%	99.90%	99.86%	99.90%	A
no. aberr.	0	1	3	2	3	4	3	
	identity	100.00%	99.86%	99.97%	99.93%	99.90%	99.93%	B
	no. aberr.	0	4	1	2	3	2	
		identity	100.00%	99.90%	99.86%	99.83%	99.86%	C
		no. aberr.	0	3	4	5	4	
			identity	100.00%	99.97%	99.93%	99.97%	D
			no. aberr.	0	1	2	1	
				identity	100.00%	99.97%	100.00%	E
				no. aberr.	0	1	0	
					identity	100.00%	99.97%	F
					no. aberr.	0	1	
						identity	100.00%	G
						no. aberr.	0	

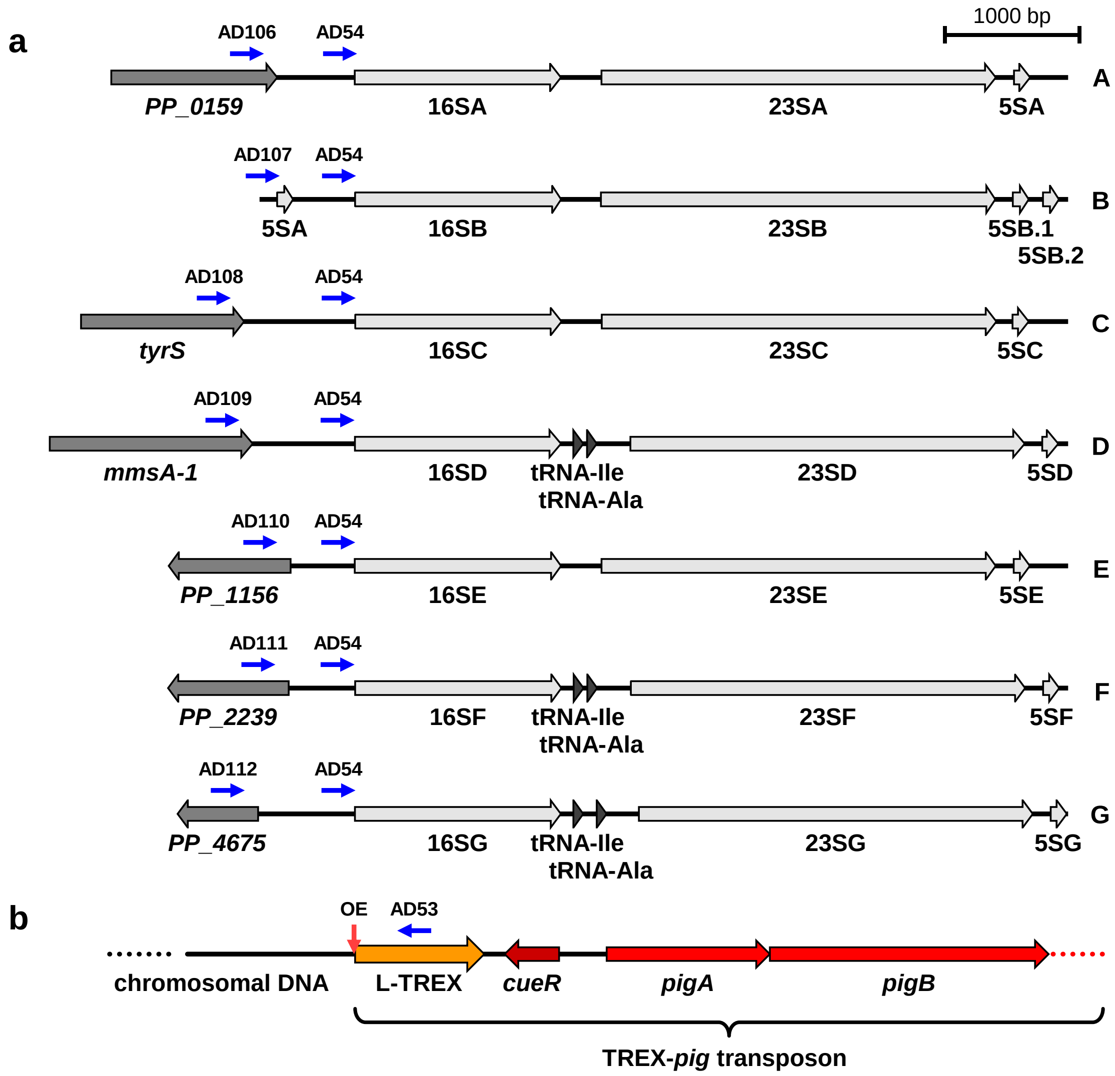

Figure S1. Primer binding sites for PCR screens of TREX-*pig* transposon insertion in rDNA of *P. putida* strains pig-r1 to pig-r52. (**a**) Schematic representation of the *rrn* operons (A-G) of *P. putida* KT2440. (**b**) Schematic of the TREX-*pig* transposon, integrated in the chromosomal DNA (not to scale).

The forward primer AD54 binds in the conserved sequence upstream of the 16S rRNA gene in any of the *rrn* operons. The reverse primer AD53 binds in the L-TREX cassette of the TREX-*pig* transposon. Use of primers AD54 and AD53 thus results in PCR product generation in case the transposon is inserted into any of the *rrn* operons. The seven forward primers AD106-112 bind specifically in the regions upstream of the conserved *rrn* promoter sequences which are individual for every *rrn* operon. Therefore, use of the seven primer pairs AD106-112 and AD53 results in PCR product generation only for one case of transposon insertion into a specific *rrn* operon.

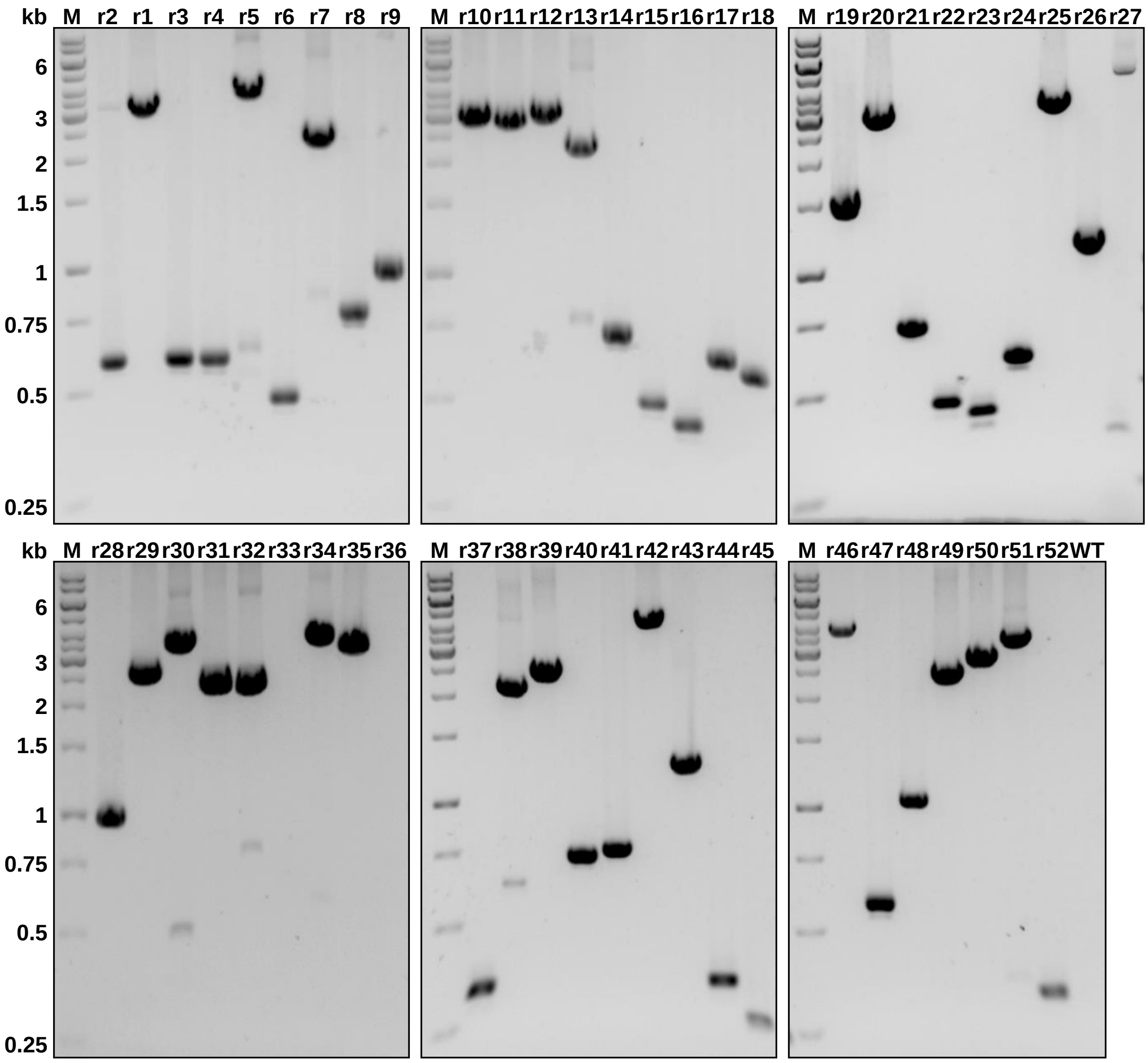

Figure S2. Results of PCR analysis for detection of TREX-*pig* transposon insertion in rDNA of *P. putida* strains pig-r1 to pig-r52. Genomic DNA *of P. putida* strains was used as template and PCR primers AD54, which binds as forward primer in the conserved sequence upstream of the 16S rRNA genes of the *rrn* operons, and AD53, which binds as reverse primer in the TREX-*pig* transposon, were employed for the analysis (see supplementary **Fig. S1**). Strains *P. putida* pig-r1 and pig-r2 were used as positive control since the insertion of the TREX-*pig* transposon in the rDNA was previously determined (Domröse et al., 2015; doi:10.3389/fmicb.2015.00972). The wildtype *P. putida* KT2440 was used as negative control. PCR products were analyzed *via* agarose gel electrophoresis using a gel with 1% agarose in 0.5x TBE-buffer (45 mM TRIS, 45 mM borate, 1 mM EDTA; Carl Roth®, Karlsruhe, Germany) in a Wide Mini-Sub Cell GT horizontal electrophoresis system (Bio-Rad Laboratories GmbH, Munich, Germany) with 135 V for 60 min with 0.5x TBE-buffer as electrophoresis buffer. An Eagle Eye II-System (Stratagene, Heidelberg, Germany) was employed for documentation of ethidium bromide-stained DNA. Strain acronyms are indicated above each lane. M, GeneRuler™ 1 kb DNA Ladder (ThermoFisher Scientific GmbH, Waltham, USA).

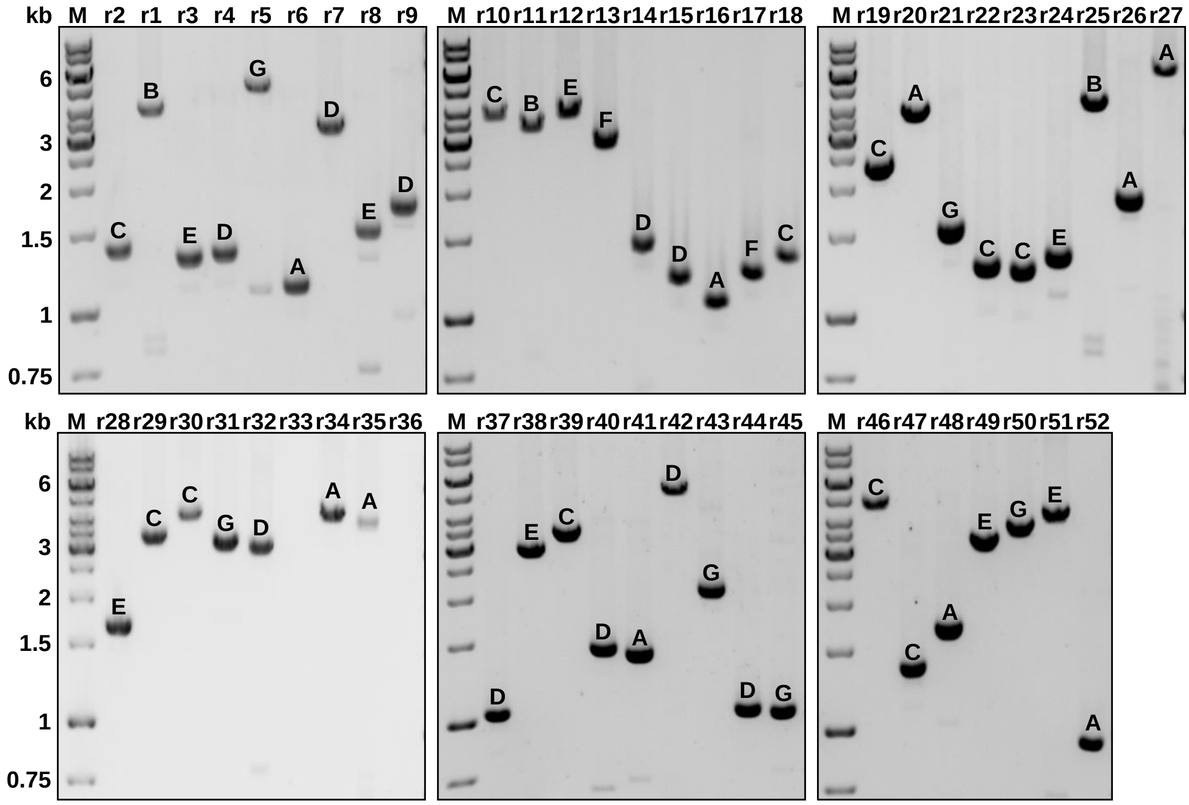

Figure S3. Summary of PCR results for detection of TREX-*pig* transposon insertion in specific *rrn* operons of *P. putida* strains pig-r1 to pig-r52. Genomic DNA *of P. putida* strains was used as template and PCR primers AD106-112, which bind as forward primer in individual sequences upstream of the *rrn* operons, and AD53, which binds as reverse primer in the TREX-*pig* transposon, were employed for the analysis (see supplementary **Fig. S1**). PCR products were analyzed via agarose gel electrophoresis using a gel with 1% agarose in 0.5x TBE-buffer (45 mM TRIS, 45 mM borate, 1 mM EDTA; Carl Roth®, Karlsruhe, Germany) in a Wide Mini-Sub Cell GT horizontal electrophoresis system (Bio-Rad Laboratories GmbH, Munich, Germany) with 135 V for 60 min with 0.5x TBE-buffer as electrophoresis buffer. An Eagle Eye II-System (Stratagene, Heidelberg, Germany) was employed for documentation of ethidium bromide-stained DNA. For each strain, only one of the seven PCR primer combinations resulted in PCR product, which is shown in the figure. The thereby identified *rrn* operons where the transposon was detected are indicated (A-G). Strain acronyms are indicated above each lane. M, GeneRuler™ 1 kb DNA Ladder (ThermoFisher Scientific GmbH, Waltham, USA).

Table S2. Chromosomal insertion loci in prodigiosin producing strains *P. putida* pig-r1 to pig-r52. The Pseudomonas Genome Database was employed for characterization of insertion loci in the chromosome of *P. putida* KT2440. Annotations are based on the file '*Pseudomonas putida* KT2440 (TIGR)' corresponding to the NCBI Genbank complete genome sequence with the accession number AE015451.2.

strain	chromosomal insertion loci of the TREX-*pig* transposon in *P. putida* KT2440 assigned *via* the Pseudomonas Genome Database			
pig-	locus tag[1]	sequence 5' → 3' upstream of the OE L-TREX[2]	position in genome[3]	strand[4]
r1	PP_23SB	ACGATGTGGGAAGGCTTAGAC	179672 / 179673	+
r2	PP_16SC	AACTGAGACACGGTCCAGACT	525267 / 525268	+
r3	PP_16SE	AACTGAGACACGGTCCAGACT	1325821 / 1325822	+
r4	PP_16SD	AACTGAGACACGGTCCAGACT	698142 / 698143	+
r5	PP_23SG	AACCGTACCCCAAACCGACAC	5308981 / 5308980	-
r6	PP_16SA	AAAGCAGGGGACCTTCGGGCC	171582 / 171583	+
r7	PP_23SD	GGAACCCAGCCAGCATAAGCT	699994 / 699995	+
r8	PP_16SE	AGAATAAGCACCGGCTAACTC	1325994 / 1325995	+
r9	PP_16SD	ATAGGAAGGAACACCAGTGGC	698528 / 698529	+
r10	PP_23SC	ACGATGTGGGAAGGCTTAGAC	527802 / 527803	+
r11	PP_23SB	TTAGGTAACACTGACTGGAGG	179352 / 179353	+
r12	PP_23SE	GCTCAAACCATGCACCGAAGC	1328466 / 1328467	+
r13	PP_23SF	AGCCTGCGATAAGCTTTGGGG	2550799 / 2550800	+
r14	PP_16SD	CAGTAAGTTAATACCTTGCTG	698278 / 698279	+
r15	PP_16SD	GCTATCAGATGAGCCTAGGTC	698042 / 698043	+
r16	PP_16SA	TTTCGAAAGGAACGCTAATAC	171542 / 171543	+
r17	PP_16SF	TGGGCGAAAGCCTGATCCAGC	2549063 / 2549064	+
r18	PP_16SC	ACACGGTCCAGACTCCTACGG	525274 / 525275	+
r19	PP_16SC	AGCCGCGAGGTGGAGCTAATC	526210 / 526211	+
r20	PP_23SA	TACAAGTGCCGAGCATGGGAG	174135 / 174136	+
r21	PP_16SG	GAAGGGCAGTAAGTTAATACC	5312233 / 5312232	-
r22	PP_16SC	CTACGGGAGAAAGCAGGGGAC	525133 / 525134	+
r23	PP_16SC	TTTCGAAAGGAACGCTAATAC	525102 / 525103	+
r24	PP_16SE	AACTGAGACACGGTCCAGACT	1325821 / 1325822	+
r25	PP_23SB	ACGATGTGGGAAGGCTTAGAC	179672 / 179673	+
r26	PP_16SA	TAAACGATGTCAACTAGCCGT	172200 / 172201	+
r27	PP_23SA	AACTTGCCTCAAGATGAGATC	175976 / 175977	+
r28	PP_16SE	GGCGAAGGCGACCACCTGGAC	1326225 / 1326226	+
r29	PP_23SC	GCGAGCTTAACCGAATAGGGG	527373 / 527374	+
r30	PP_23SC	TCCAGGTTTAAGGTGGTAGGC	528232 / 528233	+
r31	PP_23SG	AGCCTGCGATAAGCTTTGGGG	5310507 / 5310506	-
r32	PP_23SD	AGCCTGCGATAAGCTTTGGGG	699933 / 699934	+
r34	PP_23SA	AGGCGACGAAGTGGTTGATAC	174750 / 174751	+
r35	PP_23SA	ACGATGTGGGAAGGCTTAGAC	174242 / 174243	+
r37	PP_16SD	GCAGGCCTAACACATGCAAGT	697870 / 697871	+
r38	PP_23SE	TCTGAATGGGGGAACCCAGCC	1327450 / 1327451	+
r39	PP_23SC	CGAAAAGAACCCCGGAGAGGG	527246 / 527249	+
r40	PP_16SD	TAATACCTTGCTGTCTTGACG	698286 / 698287	+
r41	PP_16SA	AGAATAAGCACCGGCTAACTC	1325994 / 1325995	+
r42	PP_23SD	GATAGGTGGGAGGCTTTGAAG	701973 / 701974	+
r43	PP_16SG	TTTCCAGAGATGGATTGGTGC	5311676 / 5311675	-
r44	PP_16SD	GCAGGCCTAACACATGCAAGT	697870 / 697871	+
r45	upstream PP_16SG	TTGCTGAGCCAAGTTTAGGGT	5312720 / 5312719	-
r46	PP_23SC	AATTCCTTGTCGGGTAAGTTC	528708 / 528709	+
r47	PP_16SC	GATGATCAGTCACACTGGAAC	525249 / 525250	+
r48	PP_16SA	GGGGAGCAAACAGGATTAGAT	172160 / 172161	+
r49	PP_23SE	TGACCGATAGTGAACCAGTAC	1327768 / 1327769	+
r50	PP_23SG	TGAAACCGTATGCGTACAAGC	5310068 / 5310067	-
r51	PP_23SE	TTAATCGACGCAGGGTTAGTC	1328651 / 1328652	+
r52	PP_16SA	ACGCTGGCGGCAGGCCTAACA	171426 / 171427	+

[1] Pseudomonas Genome DB locus tag of the gene with integrated TREX-*pig* transposon.

[2] sequence immediately upstream of the outside end (OE) of the recombinant transposon.

[3] genome coordinates of the nucleotide downstream of which the transposon is integrated and of the first base of the TREX-*pig* transposon.

[4] strand of the chromosome harboring the specified tagged gene, sequence and nucleotide position.

Table S3. Summary of *P. putida* TREX-*pig* libraries, selection procedures and conducted examinations.

data set	library size[1]	criterion for selection of clones for tests[2]	positive clones based on criterion[3]	detection of rDNA insertion in selected clones[4]	investigation of clones[5]
#1: main data set (Figure 3)	50,000 clones	very intense red color	50 clones (0.1% of total library)	All clones were tested: 50 / 50 tested clones (0.1% of total library)	insertion loci and prodigiosin production investigated for all 50 strains (irrespective of specific *rrn* operon insertion) (Figure 3)
#2: supplementary data set (Figure S5)	30,000 clones	light red to intense red color	317 clones (1.1% of total library)	A sample of clones was tested: 59 / 132 tested clones (45% of tested clones) (0.5% estimated in total library)	insertion loci and prodigiosin production investigated for 19 strains (with insertion in *rrn* operon B, E, F, G) (Figure S5)

**proportion of rDNA in the *P. putida* KT2440 genome[6]**	0.25%
**proportion of *rrn* operons A, C, D in the *P. putida* KT2440 genome[6]**	0.1%

[1] libraries were constructed by integration of the TREX-*pig* transposon (based on Tn5) in *P. putida* KT2440.

[2] criteria were defined according to the research questions, i.e. aiming to find strains that showed highest production (data set #1) or to find strains with insertions in the *rrn* operons B, E, F and G.

[3] number of all clones that were found in the libraries based on the respective selection criteria.

[4] all clones that were selected based on the applied criteria (data set #1) or a representative subset (data set #2, as indicated) were tested for transposon insertion into the rDNA by PCR-based assays.

[5] determination of prodigiosin production levels as well as transposon insertion site mapping was conducted for all clones with confirmed transposon insertion into the rDNA (data set #1) or the subset of clones with confirmed transposon insertion in the *rrn* operons B, E, F and G (dataset #2, as indicated).

[6] for comparison to the fraction of clones that were confirmed to carry the transposon in the rDNA (data set #1: 0.1% of total library; data set #2: 0.5% of total library), the fraction of the *P. putida* KT2440 chromosome (two strands à 6,181,873 bp) encoding for 16S and 23S rRNA (1522 bp and 2902 bp, respectively) in all seven *rrn* operons (7 x 4,424 = 30,968 bp) or in the *rrn* operons A, C and D (3 x 4,424 = 13,272 bp), which are overrepresented in data set #1, is given. [The short 5S rRNA-encoding genes (120 bp) were not taken into account, as no insertions into this gene were observed in this study.]

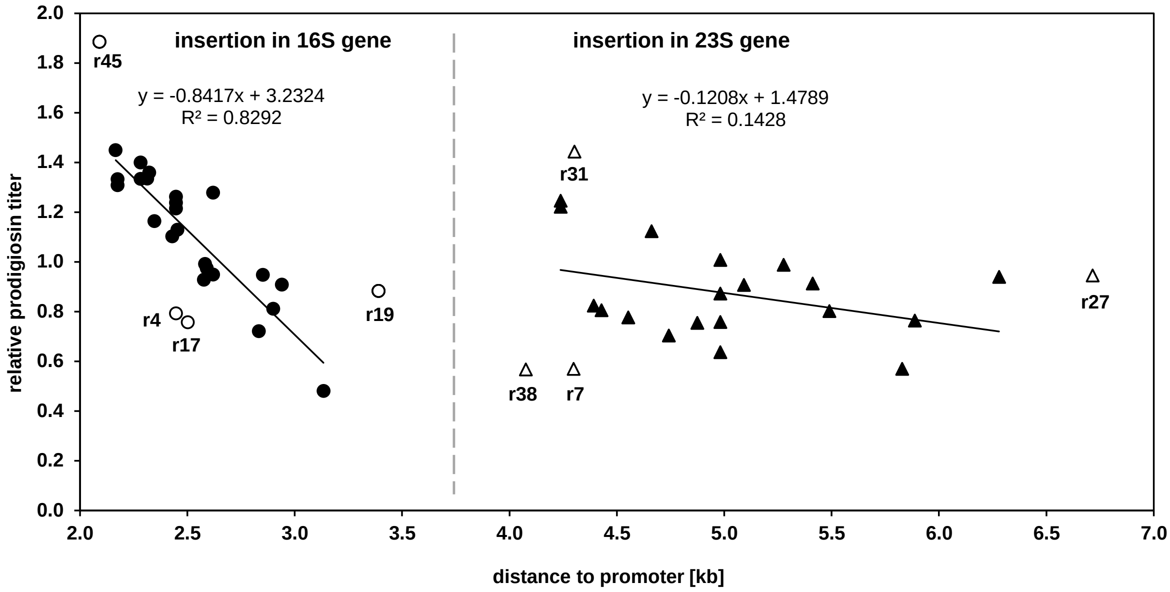

Figure S4. Correlation of distances from *rrn* promoters to *pig* genes with prodigiosin production in *P. putida* pig-r1 to pig-r52. Prodigiosin titers were normalized *rrn* operon-wise: Mean production titers, which were determined for each group of strains carrying the TREX-*pig* transposon in the same *rrn* operon, were used to normalize prodigiosin production for all strains with gene insertion in the respective *rrn* operons. Relative prodigiosin production and distances to the *rrn* promoter (as also shown in **Fig. 3**) up to 3.4 kb (insertion in the 16S rRNA gene) were included in one linear fit, excluding outliers pig-r4, -r17, -r19 and –r45. Relative prodigiosin production and distances to the *rrn* promoter from 4.1 kb (insertion in the 23S rRNA gene) were included in another linear fit, excluding outliers pig-r38, -r7, -r31 and –r27.

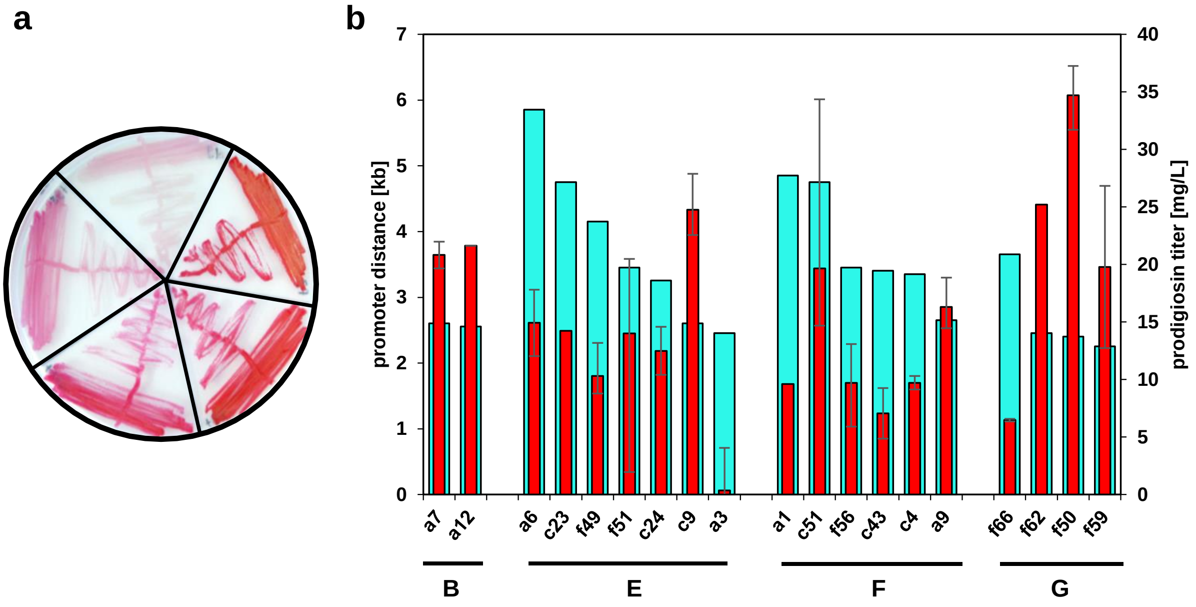

Figure S5. Correlation of rDNA insertion loci and promoter distances with prodigiosin titers in a second library of *P. putida* strains carrying the TREX-*pig* transposon. A library of ca. 30,000 clones carrying the TREX-*pig* transposon was specifically screened by phenotype and PCR analysis for transposon insertion into the *rrn* operons B, E, F and G. (**a**) Examples of light pinkish to red color phenotypes based on which clones were pre-selected for PCR analysis. In the library, a total of 317 clones exhibited one of these phenotypes. (**b**) In 132 tested clones, 59 were tested positive for general rDNA insertions, and a set of 19 strains was identified with insertion loci in the targeted operons (B, E, F, G). For these, promoter distances were estimated based on PCR products and prodigiosin production in liquid medium was determined. Turquoise bars indicate the distance of the translation start of *pigA* to the *rrn* promoter [left axis, kb]. Red bars represent prodigiosin titers [right axis, mg/L]. Designations of strains (*P. putida* pig-a1 to pig-f59) are given below bars. Results are grouped by *rrn* operons in which the *pig* genes were inserted (B, E, F, G), and within operons sorted by the promoter distance. Prodigiosin titers are median values of duplicate or quadruplicate measurements with the maximal and minimal values indicated as errors.

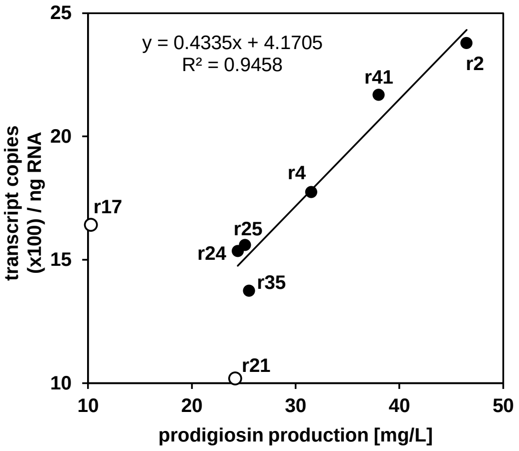

Figure S6. Correlation of *pig* transcript levels to prodigiosin production in *P. putida* pig-r strains. Prodigiosin titers [mg/L] (as also shown in **Fig. 3** and **Fig. 4** in the manuscript) and transcript copy numbers of *pigN* (as also shown in **Fig. 4**) of strains *P. putida* pig-r24, -r25, -r35, -r4, -r41 and -r2 were included in a linear fit; outliers pig-r17 and -r21 were not included in the fit.

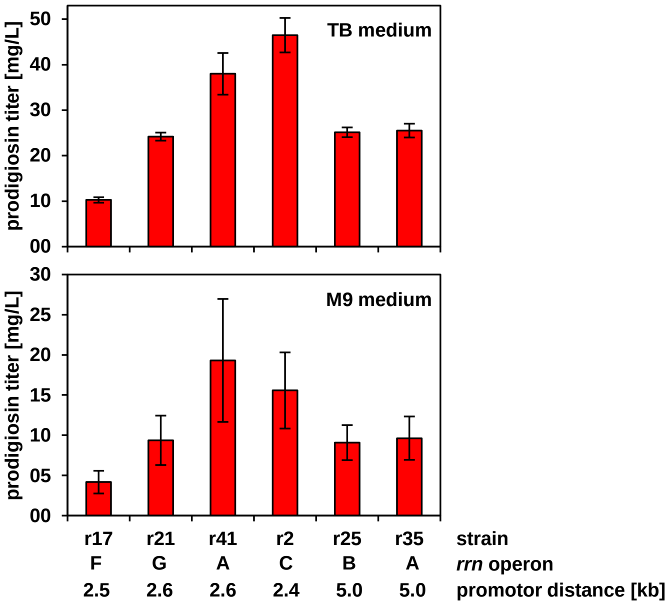

Figure S7. Comparison of prodigiosin production and *pig* gene expression in TB and M9 medium. Data on cultivation in TB medium (as also shown in **Fig. 4** of this manuscript) is compared to data obtained upon cultivation in M9 minimal medium. After pre-cultivation in the respective medium, test cultures of the *P. putida* strains were inoculated to a starting $OD_{650} = 0.05$ and samples were extracted after 24 h, as described for TB medium in the methods section. Names of *P. putida* strains and TREX-*pig* insertion loci specifications, i.e. *rrn* operons and distances between the *rrn* promoter and *pigA* start, are indicated. In both media, strains grouped in the same pattern as low-level producers (pig-r17), intermediate producers (pig-r21, -r25, -r35) and high-level producers (pig-r41, -r2). Prodigiosin titers are mean values of triplicate measurements with the respective standard deviation.

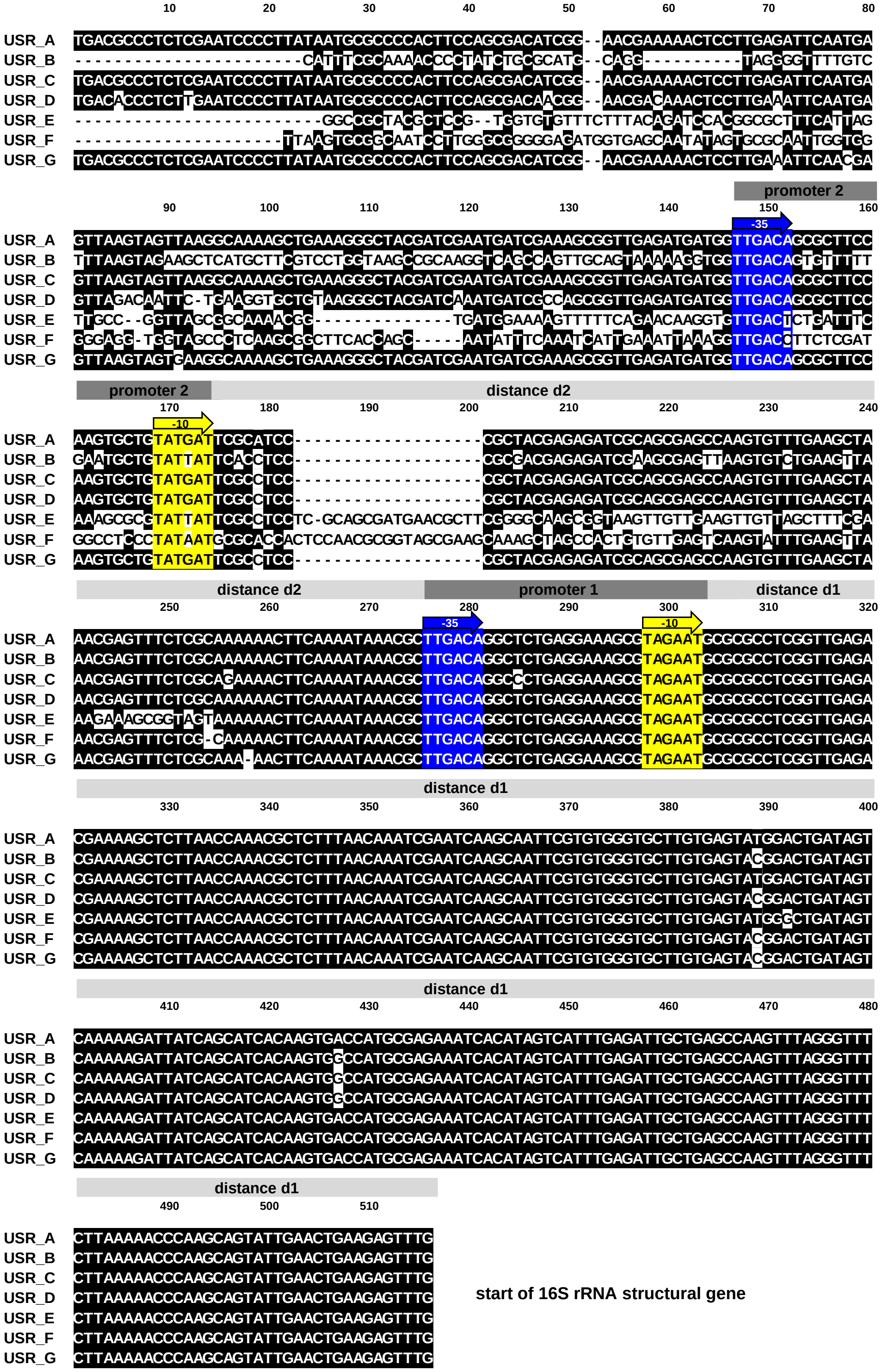

Figure S8. Sequence alignment of promoter regions upstream of the seven *rrn* operons of *P. putida* KT2440. Upstream regions (USR) of the 16S rRNA structural genes of *rrn* operons A-G were compared using ClustalOmega for multiple sequence alignment using the USR of *rrnA* as a reference. The regions containing the two promoters and regions here designated as distances d1 and d2 as well as the start of the 16S rRNA structural genes are indicated. Consensus sequences are shown in white letters on black background; aberrations are highlighted with black letters on white background. The -10 and -35 consensus sequences are highlighted yellow and blue, respectively.

Table S4. Oligonucleotides used in this study. Binding sites of PCR primers are depicted in **supplementary Fig. S1**. Binding sites of oligonucleotides used for sequencing are shown in **Fig. 2** of this manuscript.

#	application	nucleotide sequence (5'→3')
**primers for PCR screens**		
**AD53**	reverse primer binding in L-TREX	TGGCTACCCGTGATATTGCTGAAGAGCTTG
**AD54**	forward primer binding upstream of any rRNA operon	GTCATTTGAGATTGCTGAGCCAAGTTTAGGG
**AD106**	forward primer binding specifically upstream of rRNAA	TACGGTTGTCGGAGACGCCAGTGGAGTATC
**AD107**	forward primer binding specifically upstream of rRNAB	CCCATCCCGAACTCAGTAGTGAAACGATGC
**AD108**	forward primer binding specifically upstream of rRNAC	AAGAAGTCGTTTGGCCGGGTTACCCTCAAG
**AD109**	forward primer binding specifically upstream of rRNAD	CATGAAGCGTCGCAGTTTGCATTCCCTAGC
**AD110**	forward primer binding specifically upstream of rRNAE	AGGATCACTGCAAGAAAGCCCGCACTGTAG
**AD111**	forward primer binding specifically upstream of rRNAF	AGCAGCATGATGATGCTGGCGAAGATCAGG
**AD112**	forward primer binding specifically upstream of rRNAG	CTTGGGTTGGGCTTGTTGCATTTCCTGTGG
**oligonucleotides used for sequencing**		
**AD134**	sequencing primer for rRNA operon-specific upstream regions	TTTCCTCAGAGCCTGTCAAG
**AD63**	sequencing primer for transition of L-TREX to chromosome	CCCGTGATATTGCTGAAGAG
**primers for RT-qPCR**		
**AD153**	forward qPCR primer for *pigN*	TACCTGATAGGCACGCTGTT
**AD154**	reverse qPCR primer for *pigN*	TTGTTCGGATCCTGTTTGAA
**AD155**	forward qPCR primer for *rpoD*	TCGCCAAGAAGTACACCAAC
**AD156**	reverse qPCR primer for *rpoD*	TTTCATCAGACCGATGTTGC
